# Diagnostic efficiency of existing guidelines and the AI-SONIC™ artificial intelligence for ultrasound-based risk assessment of thyroid nodules

**DOI:** 10.3389/fendo.2023.1116550

**Published:** 2023-02-15

**Authors:** Linxin Yang, Ning Lin, Mingyan Wang, Gaofang Chen

**Affiliations:** ^1^Department of Ultrasound, Shengli Clinical Medical College of Fujian Medical University, Fuzhou, China; ^2^Department of Ultrasound, Fujian Provincial Hospital, Fuzhou, China

**Keywords:** thyroid nodule, papillary thyroid carcinoma, medullary thyroid carcinoma, ultrasonography, artificial intelligence

## Abstract

**Introduction:**

The thyroid ultrasound guidelines include the American College of Radiology Thyroid Imaging Reporting and Data System, Chinese-Thyroid Imaging Reporting and Data System, Korean Society of Thyroid Radiology, European-Thyroid Imaging Reporting and Data System, American Thyroid Association, and American Association of Clinical Endocrinologists/American College of Endocrinology/Associazione Medici Endocrinologi guidelines. This study aimed to compare the efficiency of the six ultrasound guidelines vs. an artificial intelligence system (AI-SONICTM) in differentiating thyroid nodules, especially medullary thyroid carcinoma.

**Methods:**

This retrospective study included patients with medullary thyroid carcinoma, papillary thyroid carcinoma, or benign nodules who underwent nodule resection between May 2010 and April 2020 at one hospital. The diagnostic efficacy of the seven diagnostic tools was evaluated using the receiver operator characteristic curves.

**Results:**

Finally, 432 patients with 450 nodules were included for analysis. The American Association of Clinical Endocrinologists/American College of Endocrinology/Associazione Medici Endocrinologi guidelines had the best sensitivity (88.1%) and negative predictive value (78.6%) for differentiating papillary thyroid carcinoma or medullary thyroid carcinoma vs. benign nodules, while the Korean Society of Thyroid Radiology guidelines had the best specificity (85.6%) and positive predictive value (89.6%), and the American Thyroid Association guidelines had the best accuracy (83.7%). When assessing medullary thyroid carcinoma, the American Thyroid Association guidelines had the highest area under the curve (0.78), the American College of Radiology Thyroid Imaging Reporting and Data System guidelines had the best sensitivity (90.2%), and negative predictive value (91.8%), and AI-SONICTM had the best specificity (85.6%) and positive predictive value (67.5%). The Chinese-Thyroid Imaging Reporting and Data System guidelines had the best under the curve (0.86) in diagnosing malignant tumors vs. benign tumors, followed by the American Thyroid Association and Korean Society of Thyroid Radiology guidelines. The best positive likelihood ratios were achieved by the Korean Society of Thyroid Radiology guidelines and AI-SONICTM (both 5.37). The best negative likelihood ratio was achieved by the American Association of Clinical Endocrinologists/American College of Endocrinology/Associazione Medici Endocrinologi guidelines (0.17). The highest diagnostic odds ratio was achieved by the American Thyroid Association guidelines (24.78).

**Discussion:**

All six guidelines and the AI-SONICTM system had satisfactory value in differentiating benign vs. malignant thyroid nodules.

## 1 Introduction

Thyroid nodules can be detected by ultrasound (US) in 19%-67% of the general population. Most detected nodules are benign and without clinical significance, but up to 7%-15% are malignant (1). Among all cancers worldwide, the incidence of thyroid carcinoma ranks 11^th^ in all patients and sixth in females (2, 3). The incidence of medullary thyroid carcinoma (MTC) represents <5% of all thyroid carcinomas (4, 5).

The risk stratification system of the American College of Radiology Thyroid Imaging Reporting and Data System (ACR-TIRADS) was proposed in 2017 (6). Its main purpose is to assess the risk of malignancy and help decide whether a US-guided fine-needle aspiration (US-FNA) and follow-up should be performed (7, 8). Owing to the mismatch of classification systems used by different hospitals, the TI-RADS is affected by low diagnostic specificity (9) and is difficult to use in everyday clinical work (10, 11). In search of better diagnostic options, local institutions worldwide have issued additional guidelines: Chinese-TIRADS (C-TIRADS) (8), European-TIRADS (EU-TIRADS) (12), and other diagnostically-modified TIRADS (11). Other diagnostic guidelines include the Korean Society of Thyroid Radiology (KSThR) guidelines published in 2017 (13), the American Thyroid Association (ATA) guidelines published in 2015 (14), and the American Association of Clinical Endocrinologists (AACE), the American College of Endocrinology (ACE) and the Associazione Medici Endocrinologi (AME) guidelines published in 2016 (15). In addition, diagnostic imaging has gradually begun to take advantage of artificial intelligence (AI) and machine learning in applying TI-RADS (16–19).

Nevertheless, the existing TIRADS mainly focuses on papillary thyroid carcinoma (PTC), with less attention being paid to MTC. Although MTC accounts for only <5% of thyroid carcinomas, MTC is characterized by a rapid progression and a worse prognosis than PTC, resulting in approximately 13% of all thyroid cancer-related deaths (3, 20). In addition, MTC has a low sensitivity to chemoradiotherapy and a high recurrence rate postoperatively (21). Thus, it is important to identify MTC early to improve patient outcomes. US is the first option for the early screening of thyroid nodules due to its safety, convenience, and good display ability of small lesions (1).

Different treatments and prognoses of MTC and PTC substantiate the need for more precise tools to diagnose them adequately as soon as possible. In addition, selecting the best diagnostic guidelines remains controversial, and applying such guidelines relies on local imaging practices and sonographers’ experience, introducing some subjectivity in the assessment. The AI-SONIC™ artificial intelligence assistant diagnostic system (Zhejiang Demetics Medical Technology Co., Zhejiang, China) is diagnostic tool able to sort US images automatically (22–25); it was developed by deep learning from the US image and pathological data of >200,000 thyroid nodules (26). Still, the comparisons between the AI-SONIC™ and the available guidelines are unknown.

Therefore, the purpose of this study was to analyze the diagnostic value of the six US guidelines and an artificial intelligence system (AI-SONIC™) in differentiating malignant vs. benign thyroid nodules.

## 2 Materials and methods

### 2.1 Patients

This retrospective study included consecutive patients who underwent thyroid nodule resection between May 2010 and April 2020 at Fujian Provincial Hospital. This study was approved by the ethics committee of Fujian Provincial Hospital. The requirement for informed consent was waived by the committee because of the retrospective nature of the study. A 10-year interval was selected in the system, and the patients who met the inclusion criteria were collected. The inclusion criteria were 1) underwent US examination at the authors’ hospital before thyroid nodule resection and 2) with a definite postoperative pathological diagnosis. Patients with poor-quality US images (image blur or non-standard image acquisition) or incomplete data (missing images, missing reports, non-standard reports, or surgery performed at another hospital) were excluded. After the screening of US image quality and clinical data, 432 patients could be included.

### 2.2 Data collection

The patients were scanned using similar equipment by operators with at least 8 years of experience. Thyroid US images were obtained from the Picture Archiving and Communication System (PACS) of the medical records. All features were reviewed by three sonographers. In case of disagreement, the features were confirmed by three radiologists. All features were re-analyzed by a chief physician with more than 20 years of experience to make a classification. All image reviewers were blind to the initial diagnosis. The evaluation for each case was performed according to the following guidelines: ACR TI-RADS (6), ATA (14), AACE/ACE/AME (15), KSThR (13), EU-TIRADS (12), and C-TIRADS (8).

Each patient was also analyzed using the AI-SONIC™ system. The AI-SONIC™ system is automatic and requires minimal user intervention. Two sonographers performed image analysis using the AI-SONIC™ system. First, 2D static images in the DICOM format were imported into the AI-SONIC™ system, which automatically identified and located the thyroid nodule lesions in the image, outlined the edges of the lesions, automatically interpreted the features of the lesions in the edge line, and scored the nodules. The nodules were considered mildly suspicious and benign when the score ranged from 0 to 0.4. When the score ranged from 0.41 to 0.99, the nodules were suspected to be malignant (0.41 to 0.60 was moderately suspicious, and 0.65 to 0.99 was highly suspicious). In a small number of patients, the automatic identification of the thyroid nodules by the AI was not accurate enough (e.g., nodule volume was too large and occupied the entire image and beyond, nodules with fuzzy boundaries, internal echo was extremely uneven, nodules breakthrough thyroid nodules coated edge were uneven, thyroid parenchyma echo, multiple adjacent nodules, and internal calcification in the acoustic shadow behind the lesion). At this point, the radiologist manually re-delineated the target nodule area, and the AI detection system automatically gave a new score based on the manually delineated nodule area.

One or two nodules were evaluated per patient. For patients with multiple nodules, the physicians in charge of data collection screened the nodules (these physicians were not participating in the subsequent interpretation of the nodules) and selected the nodules in which the US pictures and descriptions accurately corresponded to the pathological descriptions in the study. The nodules in which the US and pathological descriptions could not be matched were excluded from the study.

The following features were examined. Structure: cystic, solid, and cystic and solid (cystic: solid component <5%; solid: cystic component <5%; cystic and solid: solid component 5%-95%). Echo texture: uniform and uneven (divided according to whether the echo inside the nodule is uniform). Internal echo: very hypoechoic, hypoechoic, isoechoic, and hyperechoic (if the echo is lower than the banded muscle in the neck, it is very hypoechoic; if the echo is lower than the thyroid parenchyma, it is hypoechoic; echo equal to the thyroid parenchyma echo is isoechoic; if the echo is higher than the thyroid parenchyma echo, it is hyperechoic). Boundary: clear or fuzzy (divided according to whether the boundary between the nodule and thyroid parenchyma is clear). Edge: regular and irregular (regular means that the nodule is round or oval; irregular means that the nodule edge is lobulated or needle tip). Hyperechoic foci: coarse calcification, microcalcification, marginal/eggshell calcification, interrupted and prominent marginal calcification, and comet tail sign (coarse calcification refers to rough hyperechoic nodules with an internal diameter >1 mm, which may be accompanied by sound shadow; microcalcification refers to the scattered small strong echo with diameter <1 mm in the nodule; marginal/eggshell calcification refers to part or all of the strong echo located at the edge of the nodule; marginal calcification interruption and protrusion refer to incomplete marginal calcification with its contents protruding at the interruption; the comet tail sign refers to the point like strong echo followed by a V-shaped echo about 1-mm deep). Acoustic halo: whether the low loop vocal cord at the edge of the nodule exists. Aspect ratio (evaluated on the transverse or longitudinal section): including >1 and <1 (aspect ratio >1 means that the anterior-posterior diameter of the nodule is equal or greater than the transverse or longitudinal diameter; aspect ratio <1 means that the anterior-posterior diameter of the nodule is smaller than the transverse or longitudinal diameter. Extrathyroid extension: including contiguous capsule, invasion of the capsule, and destruction of surrounding (contiguous capsule refers to the nodule adjacent to thyroid capsule; invasion of capsule refers to the continuous interruption at the intersection of thyroid capsule and nodule; destruction of peripheral finger nodules, a breakthrough of capsule and invasion of surrounding tissues). Blood flow: the blood flow around and inside the nodule was recorded as no, small, and rich blood flow. Suspected metastatic lymph nodes: the presence or absence of suspected lymph nodes in the neck.

### 2.3 Statistical analysis

SPSS 19.0 (IBM, Armonk, NY, USA) was used for statistical analysis. The categorical variables were expressed as n (%) and analyzed using the chi-square test or Fisher’s exact test. The continuous variables were expressed as means ± standard deviations or medians (ranges) and analyzed using Student’s t-test or the Mann-Whitney U-test. A receiver-operating characteristic (ROC) curve was used to evaluate the guidelines’ diagnostic values and AI-SONIC™ system. Sensitivity, specificity, positive and negative likelihood ratios (PLR and NLR), positive predictive value (PPV), negative predictive value (NPV), and diagnostic odds ratio (DOR) were calculated for each diagnostic tool. Two-sided *P*-values <0.05 were considered statistically significant.

## 3 Results

### 3.1 Characteristics of the patients and nodules

Initially, 433 patients were included, but one patient with both PTC and MTC was excluded. Therefore, 432 patients (64 in the MTC group, 194 in the PTC group, and 174 in the benign nodule group) with 450 nodules (80 in the MTC group, 195 in the PTC group, and 175 in the benign nodule group) were included in this study. The pathological types of benign nodules included nodular goiter (n=147), follicular tumor (n=14), and adenoma (n=14). As shown in **Table 1**, the groups were comparable regarding age, but the proportion of males in the MTC group was higher (40.6%) compared with the PTC (20.6%) and benign nodule (26.4%) groups. The size of benign nodules was the largest, followed by MTC and PTC nodules.

**Table 1:** Baseline characteristics of patients.

Feature	Medullary thyroid carcinoma (*n* = 80)	Papillary thyroid carcinoma (*n* = 195)	Benign nodules (*n* = 175)	*P*
Age (years)	49.2 ± 12.1	46.8 ± 12.0	48.3 ± 12.6	0.285
Sex (male)	26 (40.6%)	40 (20.6%)	46 (26.44%)	0.007
Nodule location*				
Upper	43 (53.8%)	68 (34.9%)	60 (34.3%)	0.006
Middle	56 (70.0%)	85 (43.6%)	132 (75.4%)	< 0.001
Lower	29 (36.3%)	63 (32.3%)	124 (70.9%)	< 0.001
Isthmus	2 (2.5%)	12 (6.2%)	10 (5.7%)	0.453
Size (mm)				
Length	16.9 (10.0, 30.4)	8.7 (5.7, 15.9)	28.9 (17.5, 38.4)	< 0.001
Width	13.8 (7.7, 27.0)	7.9 (5.4, 13.2)	22.3 (14.9, 29.7)	< 0.001
Height	10.0 (6.9, 19.7)	8.5 (5.9, 11.6)	16.6 (11.5, 22.1)	< 0.001

* If a nodule was located at the upper and middle third of the thyroid, its location was recorded as upper and middle.

The location of the nodules was assessed. Most nodules located in the lower 1/3 of the lateral lobe were benign (70.9%, compared with 36.3% in MTC and 32.3% in PTC, *P* < 0.001), while nodules located in the upper 1/3 were more likely to be MTC (53.8%, compared with 34.9% in PTC and 34.3% in benign, *P* = 0.006).

### 3.2 Ultrasound features

Most US features differed significantly among the groups, especially between malignant and benign nodules. There were statistically significant differences between the MTC and the benign groups regarding the structure, echo, external thyroid expansion, suspicious lymph nodes, peripheral blood flow, and internal blood flow (all P<0.01, normalized residual >2 or <-2 after adjustment) (**Supplementary Table S1**). There were no statistically significant differences regarding the other features. There were also some significant differences noted between the MTC and PTC features. In particular, hyper- or isoechoic nodules were described more often in MTC (20.0% compared to 11.8% in PTC, *P* < 0.001). PTC nodules were more likely to have a lobulated margin (83.1%) compared with MTC (50.0%) and benign lesions (12.6%). The most notable feature of MTC was increased blood flow, both within the nodules (63.8%, compared with 19.0% in PTC and 40.6% in benign lesions, *P* < 0.001) and around the nodules (57.5%, compared with 21.5% in PTC and 50.3% in benign lesions, *P* < 0.001).

### 3.3 Lesion classification using the guidelines

Five nodules in the MTC group, 17 in the PTC group, and 22 in the benign group did not fit any ATA category. The results of US risk classification according to the guidelines are shown in **Table 2**. Among the benign nodules, a high malignancy risk was noted in 17.7% of the cases using the ACR classification, 16.0% using ATA, 29.7% using AACE/ACE/AME, 13.7% using KSThR, 35.4% using EU-TIRADS, 12.0% using AI-assisted analysis, and 0.0% using C-TIRADS classification. Regarding the malignant nodules, all tools classified the MTC and PTC cases as being at high risk of malignancy, with no cases reported as benign.

**Table 2:** US classification of thyroid nodules according to compared guidelines.

	Classification	Medullary thyroid carcinoma (*n* = 80)	Papillary thyroid carcinoma (*n* = 195)	Benign nodules (*n* = 175)
American College of Radiology Thyroid Imaging Reporting and Data System	TR1	0	0	6 (3.4%)
	TR2	3 (3.8%)	2 (1.0%)	32 (18.3%)
	TR3	2 (2.5%)	5 (2.6%)	51 (29.1%)
	TR4	24 (30.0%)	20 (10.3%)	55 (31.4%)
	TR5	51 (63.8%)	168 (86.2%)	31 (17.7%)
American Thyroid Association	Benign	0	0	10 (5.7%)
	Extremely low	2 (2.5%)	2 (1.0%)	40 (22.9%)
	Low	3 (3.8%)	7 (3.6%)	53 (30.3%)
	Moderate	13 (16.3%)	3 (1.5%)	12 (6.9%)
	High	56 (70.0%)	167 (85.6%)	28 (16.0%)
	Unclassifiable	6 (7.5%)	16 (8.2%)	32 (18.3%)
American Association of Clinical Endocrinologists, the American College of Endocrinology, and the Associazione Medici Endocrinologi	Low	1 (1.3%)	1 (0.5%)	28 (16.0%)
	Moderate	22 (27.5%)	7 (3.6%)	95 (54.3%)
	High	57 (71.3%)	187 (95.9%)	52 (29.7%)
Korean Society of Thyroid Radiology	KTR2	0	0	9 (5.1%)
	KTR3	3 (3.8%)	2 (1.0%)	55 (31.4%)
	KTR4	26 (32.5%)	26 (13.3%)	87 (49.7%)
	KTR5	51 (63.8%)	167 (85.6%)	24 (13.7%)
European-Thyroid Imaging Reporting and Data System	EUTR2	0	0	8 (4.6%)
	EUTR3	7 (8.8%)	10 (5.1%)	81 (46.3%)
	EUTR4	14 (17.5%)	2 (1.0%)	24 (13.7%)
	EUTR5	59 (73.8%)	183 (93.9%)	62 (35.4%)
AI-SONIC™	0-0.4	29 (36.3%)	32 (16.4%)	149 (85.1%)
	0.41-0.64	2 (2.50%)	8 (4.1%)	5 (2.9%)
	0.65-0.99	49 (61.3%)	155 (79.5%)	21 (12.0%)
Chinese-Thyroid Imaging Reporting and Data System	C-TR1,2	0	0	6 (3.4%)
	C-TR3	8 (10.0%)	3 (1.5%)	71 (40.5%)
	C-TR4A	17 (21.3%)	9 (4.6%)	52 (29.7%)
	C-TR4B	18 (22.5%)	34 (17.4%)	27 (15.4%)
	C-TR4C	33 (41.3%)	136 (69.7%)	19 (10.9%)
	C-TR5	4 (5.0%)	13 (6.7%)	0

### 3.4 Diagnostic value

As shown in **Table 3**, the highest sensitivity for the evaluation of malignant vs. benign nodules was demonstrated by the AACE/ACE/AME (88.1%), C-TIRADS (85.92), and EU-TIRADS (85.6%) guidelines. The KSThR guidelines (85.6%) and AI-SONICTM (85.6%) demonstrated the highest specificity. The ATA guidelines had the best accuracy (83.7%), followed by the C-TIRADS (81.1%) and AACE/ACE/AME (81.1%) guidelines. The best PLR was achieved by the KSThR guidelines and AI-SONIC™ (both 5.37). The best NLR was achieved by the AACE/ACE/AME guidelines (0.17). The highest DOR was achieved by the ATA guidelines (24.78).

**Table 3:** Diagnostic efficiency of the six guidelines and AI system.

	Diagnostic tools	Cut-off	Sensitivity (%)	Specificity (%)	Positive predictive value (%)	Negative predictive value (%)	Accuracy (%)	Area under the curve	Positive likelihood ratio	Negative likelihood ratio	Diagnostic odds ratio
(Medullary thyroid carcinoma + papillary thyroid carcinoma) vs. benign	Chinese-Thyroid Imaging Reporting and Data System	C-TR4A	85.9	73.4	83.8	76.5	81.1	0.858	3.23	0.19	16.85
	American Thyroid Association	Moderate	85.5	80.98	88.3	76.7	83.7	0.857	4.45	0.18	24.78
	Korean Society of Thyroid Radiology	KTR4	77.6	85.6	89.6	70.5	80.7	0.847	5.37	0.26	20.53
	American College of Radiology Thyroid Imaging Reporting and Data System	TR4	80.1	80.4	86.7	71.7	80.2	0.839	4.08	0.25	16.50
	AI-SONIC™	>0.39	77.6	85.6	89.6	70.5	80.7	0.817	5.37	0.26	20.53
	European-Thyroid Imaging Reporting and Data System	EUTR4	85.6	70.5	82.3	75.3	79.8	0.802	2.90	0.20	14.17
	American Association of Clinical Endocrinologists, the American College of Endocrinology, and the Associazione Medici Endocrinologi	Moderate	88.1	69.9	82.4	78.6	81.1	0.797	2.93	0.17	17.21
Medullary thyroid carcinoma vs. benign	American Thyroid Association	Low	80.5	70.9	58.5	87.7	74.1	0.784	2.76	0.27	10.05
	American College of Radiology Thyroid Imaging Reporting and Data System	TR3	90.2	52.0	47.1	91.8	64.3	0.772	1.88	0.19	10.02
	Korean Society of Thyroid Radiology	KTR4	56.1	85.6	64.8	80.4	76.1	0.763	3.88	0.51	7.57
	Chinese-Thyroid Imaging Reporting and Data System	C-TR4A	65.9	73.4	54.0	81.9	71.0	0.759	2.48	0.47	5.32
	AI-SONIC™	>0.39	63.4	85.6	67.5	83.1	78.4	0.748	4.39	0.43	10.26
	European-Thyroid Imaging Reporting and Data System	EUTR3	84.2	59.5	49.6	88.8	67.5	0.723	2.08	0.27	7.81
	American Association of Clinical Endocrinologists, the American College of Endocrinology, and the Associazione Medici Endocrinologi	Moderate	68.3	69.9	51.9	82.3	69.4	0.713	2.27	0.45	5.01
Papillary thyroid carcinoma vs. benign	Chinese-Thyroid Imaging Reporting and Data System	C-TR4A	93.4	73.4	80.0	90.7	84.1	0.894	3.51	0.09	39.07
	American Thyroid Association	Moderate	94.4	80.8	85.4	92.4	88.2	0.885	4.92	0.07	71.44
	Korean Society of Thyroid Radiology	KTR4	85.8	85.6	87.1	84.1	85.7	0.880	5.94	0.17	35.74
	American College of Radiology Thyroid Imaging Reporting and Data System	TR4	87.8	80.4	83.6	85.3	84.3	0.864	4.47	0.15	29.48
	AI-SONIC™	>0.39	83.3	85.6	86.8	81.8	84.3	0.845	5.76	0.20	29.43
	European-Thyroid Imaging Reporting and Data System	EUTR4	94.9	70.5	78.6	92.4	83.5	0.831	3.22	0.07	44.70
	American Association of Clinical Endocrinologists, the American College of Endocrinology, and the Associazione Medici Endocrinologi	Moderate	95.4	69.9	78.3	93.1	83.5	0.829	3.17	0.07	48.59

^*^Excluding the nodules that are not classifiable using the ATA system (5 MTC,17 PTC, and 22 benign nodules).

**Figure 1:**
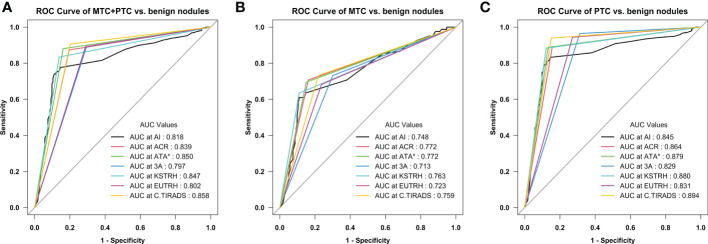
Receiver operating characteristic (ROC) curves of the seven diagnostic methods **(A)** Medullary thyroid carcinoma (MTC) + papillary thyroid carcinoma (PTC) vs. benign nodules; **(B)** MTC vs. benign nodules; **(C)** PTC vs. benign nodules. ^*^Excluding the nodules that are not classifiable using the ATA system (5 MTC, 17 PTC, and 22 benign nodules).

When comparing the diagnostic efficiency for MTC nodules, the ATA guidelines had the largest AUC (0.78), the ACR guidelines had the best sensitivity (90.2%) and NPV (91.8%), and the AI-SONIC™ system had the best specificity (85.6%), PPV (67.5%), and accuracy (78.4%). Interestingly, the AACE/ACE/AME guidelines showed the highest sensitivity for PTC (95.4%) but not for MTC (68.3%). The PLR and NLR (PLR of 5.37, NLR of 0.43) were the best for the AI-SONIC™ system. The KSThR guidelines (PLR of 3.88) were next in the ability to diagnose MTC, and the AACE/ACE/AME guidelines (NLR of 0.45) were next in the ability to exclude MTC. In the PTC group, the KSThR guidelines (PLR of 5.94) and AI-SONIC™ (PLR of 5.76) were the best, and the negative likelihood ratios of the C-TIRADS, ATA, EU-TIRADS, and AACE/ACE/AME guidelines (NLR of <0.1) were better than those of the MTC group. The ROC analysis (**Figure 1**) confirmed the high accuracy of the C-TIRADS guidelines in risk assessment for PTC nodules but not MTC.

## 4 Discussion

Although some differences in performance were observed, all six guidelines and the AI-SONIC™ system had satisfactory value in differentiating benign vs. malignant thyroid nodules.

**Figure 2:**
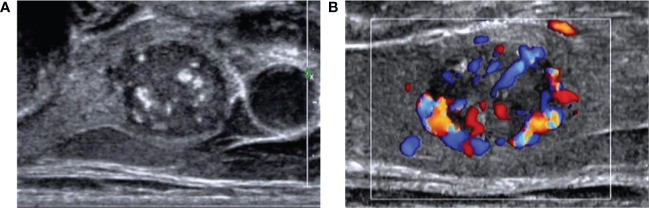
Images of a 55-year-old man with medullary thyroid carcinoma (MTC) **(A)** Ultrasound image showed a solid hypoechoic nodule with a clear boundary and lobulated margin. The aspect ratio was <1, and coarse calcification could be seen in the nodule. **(B)** Color Doppler flow imaging showed abundant blood flow around and inside the nodule.

The risk stratification systems for thyroid nodules (ACR- TIRADS and others) are often affected by a low diagnostic specificity. Recent studies noted the same limitations in applying those guidelines as in the present study (7, 10, 27–32). Significant differences were noted between MTC and PTC features: hyper- or isoechoic nodules more often described in MTC and lobulated margins in PTC. The most notable feature of MTC was the increased blood flow, both inside and around the nodules (**Figure 2**).

Peng et al. (29), in a study of 230 thyroid nodules in 2020, observed that as much as 19.6% of the malignant nodules (45 of 230) did not match any pattern of the ATA guidelines; hence, the ACR- TIRADS guidelines derived the highest diagnostic performance and the greatest level of sensitivity, compared with the AACE/ACE/AME and ATA guidelines. The present study showed comparable results in the malignant (MTC+PTC) group but not in the MTC group, where the KSThR guidelines (a classification system that Peng et al. did not evaluate) demonstrated the highest specificity and accuracy, followed by AACE/ACE/AME. A recent study by Pandya et al. (28) also examined the performance between the ACR -TIRADS and ATA guidelines and showed that both guidelines had similar diagnostic accuracies but that the ACR- TIRADS guidelines resulted in fewer nodules being recommended for immediate FNA and more nodules being recommended for imaging surveillance. In the present study, the accuracy of the ATA guidelines in assessing the risk of MTC was slightly higher than the ACR-TIRADS guidelines due to the higher specificity. Nevertheless, a higher number of unclassifiable cases in the ATA guidelines might cause problems in clinical application, especially by less experienced sonographers.

The application of the US classification systems has local specifics related to the lexicon and diagnostic traditions (11). Shen et al. (33) compared the ACR -TIRADS, ATA, EU-TIRADS, and KSThR guidelines and noted that all four risk-stratification systems had good diagnostic performances. Zhang et al. (31) compared the ACR- TIRADS, ATA, Kwak TI-RADS, and KSThR guidelines and reported that the ACR TI-RADS and Kwak TI-RADS guidelines had better diagnostic performance than the other guidelines in the malignant group. Zhang et al. (31) also noted that among the suspicious US image features, the most significant independent predictor for malignancy was hypoechogenicity. In the present study and Zhu et al. (11), hypoechogenicity was more associated with PTC than with MTC, while in MTC, hyper- or isoechoic nodules were described more often (20.0% vs. 11.8%). Conversely, due to the notable US differences, Zhu et al. (11) recently proposed a modified TI-RADS system specifically for diagnosing MTC. This modified system warrants further investigation.

Interestingly, in the recent study by Matrone et al. (34), among 152 consecutive patients with MTC, US high risk of MTC malignancy included in the EU-TIRADS, 2015 ATA, AACE/ACE-AME, ACR-TIRADS, and K-TIRADS guidelines, varied from 45.4% to 47.4%, while in the present study, it was notably higher (63.7%-73.8%). Li et al. (27) compared the value of the KWAK TI-RADS and 2015 ATA guidelines in ninety-three patients (29 with MTCs, 31 with PTCs, and 33 with thyroid adenomas). The ATA guidelines showed higher specificity and sensitivity for PTC (67.7% and 77.4%, respectively) than MTC (62.1% and 65.5%, respectively), which is consistent with the present study but also slightly lower: 80.5% and 70.9% for MTC, and 94.4% and 80.8% for PTC. Yun et al. (35) evaluated TI-RADS for MTC and reported that 95% of the nodules were classified as either highly suspicious (68%) or intermediately suspicious (26%), which were lower compared with the present study. The selection process might explain this difference based on the FNA results and the generally smaller nodules in the present study. Markedly, Hahn et al. (36) explored the diagnostic efficacy of the ATA and K-TIRADS guidelines in MTC and noted that the nodule size correlated with the diagnosis, and small MTC nodules were classified more commonly as highly suspicious. Nevertheless, the diagnostic value of the classification systems explored in the present study for MTS based on AUC were ATA (0.784) > ACR (0.772) > KSThR (0.763) > C-TIRADS (0.759) > AI (0.748) > EU-TIRADS (0.723) > AACE/ACE/AME (0.713), which is mostly consistent with the results obtained by the studies mentioned above.

**Figure 3:**
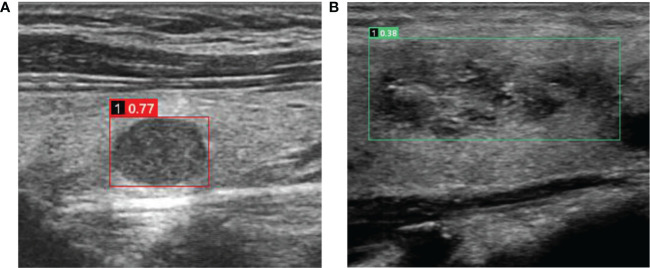
Cases in which misdiagnosis was avoided using the AI-SONIC™ system **(A)** Image of a 51-year-old man with a right thyroid nodule. The ultrasound image showed a solid hypoechoic nodule with a clear boundary and regular morphology. No obvious malignant features were observed. However, the nodule earned a score of 0.77 according to the AI-SONIC™ system, indicating a high risk of malignancy. Postoperative pathological examination confirmed the diagnosis of medullary thyroid carcinoma. **(B)** Image in a 56-year-old woman with a right thyroid nodule. The ultrasound image showed a solid isoechoic nodule with obscure boundary and irregular morphology, and the aspect ratio was <1. The internal echo was not uniform, and microcalcification could be seen. Multiple guidelines indicated a high risk of malignancy. However, the nodule earned a score of 0.38 according to the AI-SONIC™ system, indicating a high probability of a benign nodule. Postoperative pathological examination confirmed the diagnosis of nodular goiter, and interstitial fibrosis and focal cholesterol crystal could be seen.

Machine deep learning is another approach gaining popularity among scientists and physicians. The literature shows promise in applying AI technology to avoid unnecessary biopsies (37). In the study by Wang et al. (38), the performance of the YOLOv2 neural network did not significantly differ from that of the radiologists (*P* > 0.05). The AIBx algorithm, proposed by Johnson et al. (39), demonstrated better sensitivity, specificity, and PPV than the radiologists. AI-assisted TI-RADS improved specificity while maintaining sensitivity (18) and avoiding unnecessary biopsies (30). In the present study, the AI-assisted diagnostic algorithm proposed by AI-SONIC™ (Demetics) was compared with other risk assessment guidelines. The results revealed the highest specificity (85.6%), PPV (67.5%), and accuracy (78.6%) of the AI in the MTC group but average sensitivity (63.4%). In some atypical cases, the assistance of AI might avoid misdiagnosis (**Figure 3**). Image similarity AI models, as a part of diagnostic algorithms, can decrease subjectivity and increase confidence in the predictions (39). Overall, AI models could become a decision-support tool but are not yet ready to substitute for human physicians. Recently, Kang et al. (40) highlighted the usefulness of computer-aided diagnosis, especially for less experienced radiologists and especially for diagnosing PTC. Still, there are differences in performance among the various AI systems, and future studies should also examine and compare the available AI systems.

MTCs were mainly solid, and cystic changes in MTC nodules were rare (7.5%), which is consistent with the conclusions of Lee et al. (41). In benign nodules, cystic and solid structures were more common, while PTC was also mainly solid, and this feature was more significant than in MTC. MTC is mainly hypoechoic, which is consistent with the conclusions of Saller et al. (42) and Zhu (11, 43), while isoechoic, and hyperechoic nodules were more common in benign nodules, which is consistent with the pathological basis of MTC tumor cells being homogeneous, arranged in solid patches and nests, and with less stroma. PTC was also mainly hypoechoic. MTC is prone to suspicious extrathyroid extension. MTC is often invasive. Only about half (48%) of the lesions were localized, while 35% of the lesions broke through the thyroid capsule and invaded the surrounding tissues or local lymph node metastasis. Because the definition of extrathyroid extension in this study included the adjacent capsule, the occurrence rate of suspected extrathyroid extension was higher than in other similar studies. The risk of extrathyroid extension of malignant nodules was 61%, of which 31% were visible to the naked eye, and the specificity of capsule contiguity was poor. There was a >2-mm normal thyroid parenchyma between the nodules and the continuous capsule, which reduced the risk of extrathyroid extension under the microscope to <6%, and there was little or no chance of gross invasion. The suspected lymph node metastasis in the MTC group was significantly different from benign nodules and more significant than in the PTC group. MTC is a rare disease with no significant malignant features. It is difficult even for experienced sonographers to detect it early, and MTC is highly invasive and prone to lymph node metastasis (24, 42). These features are different from PTC and benign nodules, but the lymph node metastasis of MTC will affect the prognosis of patients. Therefore, it is meaningful to detect MTC lesions as soon as possible through the nodule’s characteristics before metastasis. The blood flow of MTC was abundant. The peripheral blood flow of MTC was like that of benign nodules, while the internal blood flow of MTC was significantly richer than that of benign nodules, while the peripheral and internal blood flow of PTC nodules was mostly absent. Although it differed from PTC, the blood supply of MTC overlapped with that of benign nodules, and the significance of blood flow differentiation was limited. In this study, it was found that coarse calcification was most common in MTC (33.8%), followed by PTC (28.7%), and less common in the benign group (21.7%), but the difference was not statistically significant. Microcalcifications were more common in the PTC group (49.7%), followed by the MTC group (35.0%), while it was rarer in the benign group (18.9%), but when the three groups were compared at the same time, the difference of microcalcification was only statistically significant between the PTC and benign groups. The main reason for calcification formation in MTC is that the local cancer tissue forms a calcium and phosphorus deposition microenvironment under the effect of bone matrix protein regulating cell matrix, and then amyloid surrounds the calcium and phosphorus deposition (44). Therefore, coarse calcification in MTC is slightly more common than in PTC. In this study, microcalcification and coarse calcification are common in the MTC group. Different studies have reported different types of calcifications and MTC, which might be related to the course of MTC nodules (45–47). PTC nodules also have many fibrous stromal hyperplasia, sclerosis, and rich calcareous, and coarse calcification was not rare, but microcalcification was more common in the PTC group. Microcalcification in PTC might be due to poor blood supply, and necrotic calcification occurs easily (48–50). Nevertheless, the benign nodules undergoing surgery may cause compression symptoms to the surrounding tissues and affect the appearance due to the large volume of the nodules, and the nodules have suspicious ultrasonic features, so the calcification rate of benign nodules in this study is also high. On the other hand, the benign nodules encountered in daily work might not be calcified so frequently, and this feature might still have a certain differential value.

Therefore, based on the above, regarding nodule size, it is easy to miss the diagnosis because the ultrasonic features of early MTC are not obvious. Compared with PTC, the volume of MTC is usually larger when it is found. Most studies showed that most MTCs are solid nodules with few cystic changes, while PTCs rarely have cystic changes and benign nodules often have cystic changes (42, 45, 46). Most MTCs showed low echo, followed by very hypoechoic, while hyperechoic was rare. Hypoechoic PTC nodules were also the most common, while hypoechoic, isoechoic, and hyperechoic benign nodules were not uncommon. Most authors agree that MTC nodules have clear boundaries, but some hold opposite opinions; an unclear boundary might be related to malignancy, but it might also be caused by an uneven echo of thyroid parenchyma caused by Hashimoto thyroiditis and other reasons (42, 45, 46). Of note, Hashimoto thyroiditis appears to coexist with differentiated thyroid cancers (51). There are different reports on whether MTC nodules are mainly regular in morphology. Many authors believe that MTC is mostly regular in morphology (round and quasi-round), but some scholars also found that most MTC nodules are irregular in morphology (42, 45, 46). PTC often has irregular margins, and benign nodules usually have regular margins. The aspect ratio of MTC is mainly <1, that of PTC is >1, and that of benign nodules is <1. Studies suggest that the calcification in MTC nodules is mainly amyloid deposits, so the proportion of coarse calcification is higher than that of microcalcification in MTC (42, 45, 46). In PTC, microcalcification is more common due to necrosis foci. MTC is often invasive, PTC can also have extrathyroid extension, but it is less common than MTC, while benign nodules are non-invasive. MTC often shows abundant blood flow signals in and around the nodules. PTC mainly lacks blood supply. Blood flow signals can appear in benign nodules.

The role of contrast-enhanced US (CEUS) is defined in diagnosing and managing thyroid cancer (52). CEUS is particularly useful for revealing the vascularization of tissues and is sensitive and specific for thyroid cancer (52). Still, whether CEUS could be used to differentiate PTC vs. MTC remains to be confirmed. Future studies should include multimodality US.

This study had some limitations. First, because of the low incidence of MTC, the sample size of this was relatively small, which might reduce the power of the study to some extent. Secondly, only MTCs and PTCs were included. Other malignant nodules, such as follicular carcinoma and undifferentiated carcinoma, were not included. Indeed, PTC is the most common subtype of thyroid carcinoma, but MTC has a worse prognosis. In addition, because PTC is more frequent, the classification guidelines are mainly based on the PTC features. Therefore, this study aimed to examine MTC but used PTC as controls. Future studies should also examine other subtypes. Third, only one center was involved. Since US is operator-dependent (40), the generalizability of the conclusions might be limited. Multicenter studies are necessary to address this issue. Finally, because of the retrospective nature of the present study, some US characteristic data were incomplete and could not be included in the analysis. These characteristics can be considered in future prospective studies.

In conclusion, six classification guidelines and the AI-SONIC™ system efficiently differentiate benign vs. malignant thyroid nodules. The C-TIRADS guidelines demonstrated the best performance in the authors’ setting. The ATA and ACR guidelines and the AI-SONIC™ system were effective tools for identifying MTC nodules. Thus, using more than one diagnostic tool could be associated with a more satisfactory differentiation among MTC, PTC, and benign nodules.

## Data availability statement

The original contributions presented in the study are included in the article/**Supplementary Material**. Further inquiries can be directed to the corresponding author.

## Ethics statement

The studies involving human participants were reviewed and approved by Fujian Provincial Hospital (K2019-01-051). Written informed consent for participation was not required for this study in accordance with the national legislation and the institutional requirements.

## Author contributions

Conceptualization: LY, NL. Data curation: LY, MW, GC. Formal analysis: LY, MW. Funding acquisition: NL. Investigation: LY, MW, GC. Methodology: LY, NL. Project administration: LY, MW. Resources: LY, NL. Software: LY, MW. Supervision: LY, MW, GC. Validation: LY, MW. Visualization: MW, GC. Writing-original draft: LY. Writing-review & editing: LY. All authors contributed to the article and approved the submitted version.
