# Supplementary material for: Diagnostic efficiency of existing guidelines and the AI-SONIC™ artificial intelligence for ultrasound-based risk assessment of thyroid nodules
Source: Front Endocrinol (Lausanne). 2023 Feb 15;14:1116550. doi: 10.3389/fendo.2023.1116550 (PMC9975494; doi:10.3389/fendo.2023.1116550)
Supplement: Supplementary file 1 [file Table_1.docx]

**Supplementary Table S1.** Analysis of the differences in US characteristics among the MTC, PTC, and benign groups

| Ultrasound features | | Medullary thyroid carcinoma | | Papillary thyroid carcinoma | | Benign nodules | | P |
| --- | --- | --- | --- | --- | --- | --- | --- | --- |
|  |  | n (%) | Residual | n (%) | Residual | n (%) | Residual |  |
| Structure | Cystic | 0 | -1.7 | 0 | -3.2 | 13 (7.4) | 4.6 | <0.01 |
|  | Cystic solid | 6 (7.5) | -2.1 | 3 (1.5) | -7.1 | 60 (34.3) | 8.9 |  |
|  | Solid | 74 (92.5) | 2.7 | 192 (98.5) | 8 | 102 (58.3) | -10.3 |  |
| Substantial echo uniformity | Homogeneous | 21 (26.3) | 1.4 | 52 (26.7) | 2.9 | 19 (10.9) | -4 | <0.01 |
|  | Heterogeneous | 59 (73.8) | -1.4 | 143 (73.3) | -2.9 | 156 (89.1) | 4 |  |
| Echo | Low | 47 (58.8) | 2.3 | 120 (61.5) | 5.3 | 44 (25.7) | -7.2 | <0.01 |
|  | Very low | 17 (21.3) | 1.2 | 52 (26.7) | 5 | 5 (2.9) | -6.1 |  |
|  | Equal + high | 16 (20.0) | -3.3 | 23 (11.8) | -9.4 | 122 (71.4) | 12.2 |  |
| Border | Clear | 64 (80.0) | -1.9 | 158 (81.0) | -2.9 | 167 (95.4) | 4.4 | <0.01 |
|  | Fuzziness | 16 (20.0) | 1.9 | 37 (19.0) | 2.9 | 8 (4.6) | -4.4 |  |
| Edge | Regularity | 40 (50.0) | 0 | 33 (16.9) | -12.4 | 153 (87.4) | 12.6 | <0.01 |
|  | Lobes/needle tips | 40 (50.0) | 0 | 162 (83.1) | 12.4 | 22 (12.6) | -12.6 |  |
| Aspect ratio | <1 | 64 (80.0) | 1.9 | 98 (50.3) | -8.6 | 159 (90.9) | 7.3 | <0.01 |
|  | ≥1 | 16 (20.0) | -1.9 | 97 (49.7) | 8.6 | 16 (9.1) | -7.3 |  |
| Extrathyroid extension | No | 36 (45.0) | -3 | 84 (43.1) | -6.3 | 149 (85.1) | 8.8 | <0.01 |
|  | Adjacent capsular | 22 (27.5) | 1.1 | 58 (29.7) | 3.1 | 22 (12.6) | -4.1 |  |
|  | Capsular invasion | 14 (17.5) | 1.9 | 34 (17.4) | 3.6 | 3 (1.7) | -5.1 |  |
|  | Peripheral damage | 8 (10.0) | 1.5 | 19 (9.7) | 2.7 | 1 (0.6) | -4 |  |
| Coarse calcification | No | 53 (66.3) | -1.5 | 139 (71.3) | -0.8 | 137 (78.3) | 2 | 0.099 |
|  | Yes | 27 (33.8) | 1.5 | 56 (28.7) | 0.8 | 38 (21.7) | -2 |  |
| Microcalcification | No | 52 (65.0) | 0 | 98 (50.3) | -5.7 | 142 (81.1) | 5.8 | <0.01 |
|  | Yes | 28 (35.0) | 0 | 97 (49.7) | 5.7 | 33 (18.9) | -5.8 |  |
| Suspicious lymph node | No | 53 (66.3) | -5 | 153 (78.5) | -3.1 | 174 (99.4) | 7 | <0.01 |
|  | Yes | 27 (33.8) | 5 | 42 (21.5) | 3.1 | 1 (0.6) | -7 |  |
| Peripheral blood | No | 10 (12.5) | -2 | 56 (28.7) | 3.7 | 27 (15.4) | -2.2 | <0.01 |
|  | Few | 24 (30.0) | -2.1 | 97 (49.7) | 3.6 | 60 (34.3) | -2 |  |
|  | Massive | 46 (57.5) | 3.7 | 42 (21.5) | -6.7 | 88 (50.3) | 3.9 |  |
| Internal blood | No | 14 (17.5) | -4.5 | 105 (53.9) | 5.2 | 61 (34.9) | -1.8 | <0.01 |
|  | Few | 15 (18.8) | -1.4 | 53 (27.2) | 1.1 | 43 (24.6) | 0 |  |
|  | Massive | 51 (63.8) | 5.9 | 37 (19.0) | -6.3 | 71 (40.6) | 1.9 |  |
